# Theory and practical based approach to chronic total occlusions

**DOI:** 10.1186/s12872-016-0209-3

**Published:** 2016-02-09

**Authors:** Georgios Sianos, Nikolaos V. Konstantinidis, Carlo Di Mario, Haralambos Karvounis

**Affiliations:** 1st Department of Cardiology, AHEPA University Hospital, Stilponos Kiriakidi 1, 54636 Thessaloniki, Greece; National Institute for Health Research (NIHR) Biomedical Research Unit, Royal Brompton & Harefield NHS Foundation Trust, London, United Kingdom

**Keywords:** Chronic total occlusion, CTO, Antegrade, Retrograde, Collateral circulation, Sub intimal space, True lumen re-entry

## Abstract

Coronary chronic total occlusions (CTOs) represent the most technically challenging lesion subset that interventional cardiologists face. CTOs are identified in up to one third of patients referred for coronary angiography and remain seriously undertreated with percutaneous techniques. The complexity of these procedures and the suboptimal success rates over a long period of time, along with the perception that CTOs are lesions with limited scope for recanalization, account for the underutilization of CTO Percutaneous Coronary Intervention (PCI). During the last years, dedicated groups of experts in Japan, Europe and United States fostered the development and standardization of modern CTO recanalization techniques, achieving success rates far beyond 90 %, while coping with lesions of increasing complexity. Numerous studies support the rationale of CTO revascularization following documentation of viability and ischemia in the territory distal to the CTO. Successful CTO PCI provide better tolerance in case of future acute coronary syndromes and can significantly improve angina and left ventricular function. Randomized trials are on the way to further explore the prognostic benefit of CTO revascularization. The following review reports on the theory and the most recent advances in the field of CTO recanalization, in an attempt to promote a more balanced approach in patients with chronically occluded coronary arteries.

## Background

Coronary Chronic Total Occlusions (CTOs) are identified in up to one third of patients with coronary artery disease referred for coronary angiography [1–3], with an incidence increasing with age [4]. Numerous studies support the rationale of CTO recanalization in case of documented viability and ischemia in the territory distal to the CTO. Successful CTO recanalization is related to improved survival, improvement in anginal status and left ventricular function, increased exercise tolerance, decreased need for Coronary Artery Bypass Grafting (CABG) and better tolerance of future acute coronary syndromes [5–9]. Recent series report that successful CTO Percutaneous Coronary Intervention (PCI) might also reduce the risk for arrhythmic events in patients with ischemic cardiomyopathy [10].

The success rates of operators experienced in conventional CTO PCI techniques was never greater than 60–70 % [6], considerably lower than the success rates in non-occlusive coronary artery disease. Restenosis and reocclusion were also high before the introduction of Drug Eluting Stents (DES) [11]. Randomized trials exploring the prognostic benefit of CTO revascularization have not been launced until recently and are still recruiting. No more than 10 % of all CTOs have been treated with percutaneous techniques over a long period of time, with the majority of patients primarily managed medically or referred for CABG [1, 4, 12–15]. Lesions with severe tortuosities, calcifications or large bifurcations present technical challenges, but currently dedicated centers applying new strategies achieve success rates far above 90 % [16]. The following review reexamines the evidence leading to the underutilization of CTO revascularization procedures, promoting a more balanced and proactive approach in patients suffering of this often highly disabling condition.

## Definition

A chronic total occlusion is defined as an angiographically documented or clinically suspected complete interruption of antegrade coronary flow (Thrombolysis In Myocardial Infarction-TIMI- 0 flow) of greater than 3 months standing [17]. Occasionally, bridging collaterals may provide antegrade flow to the vessel beyond the occlusion, giving the false impression of a functional sub-occlusive lesion. Careful examination of the occlusion in multiple views delineates the position of these collaterals outside the vessel architecture. The interpretation is often complicated by the presence of intraluminal microchannels, which are demonstrated pathologically in the majority of cases, and may play a role in facilitating wire crossing [18, 19], but mostly remain below the resolution of angiography (100 μm) and do not generally traverse the entire occluded segment or they violate the TIMI 0 criterion [20, 21].

## Prevalence

The prevalence of CTOs depends on the type of patients studied with an incidence ranging between 10 and 30 % of all coronary angiograms [1–3]. A history of myocardial infarction can be elicited in 30–50 % of the patients undergoing CTO PCI [6, 22], suggesting acute onset of the occlusion, whereas in the remaining patients gradual development of CTO likely occurred. Interestingly, Choi et al. utilizing magnetic resonance imaging in 170 consecutive patients with coronary CTO revealed evidence of prior MI by late gadolinium enhancement in 86 % of patients, a much higher proportion that previously recognized, with only 25 % of patients showing Q-waves on their electrocardiogram [23]. More recent reports tend to show a lower CTO prevalence, possibly explained by the universal use of primary angioplasty and early revascularization in acute coronary syndromes. Still, silent ischemia or presence of atypical symptoms misinterpreted at the time of the acute event account for the consistent persistence of CTOs in 18.4 % of patients even in the most recent series [1]. CTOs are identified in more than 50 % of patients restudied after CABG [1], with this subgroup of patients suffering lower technical success rates during native coronary occlusion recanalization compared to patients without prior CABG [24, 25]. You may expect that in patients with acute coronary syndromes CTOs are less frequent. In reality, even in patients with acute ST segment elevation myocardial infarction (STEMI), the incidence is 13 % [26], with this subgroup of patients suffering a particularly poor immediate and long-term prognosis. The presence of a CTO in a non-infarct related artery was found to be a strong and independent predictor for both early mortality (within 30 days after STEMI) and late mortality (from 30 days to 5 years after STEMI) [26, 27]. Inability to provide collaterals to the occluded vessel and, vice versa, acute impairment of preexisting collaterals from the acutely occluded vessel to the CTO jeopardizing a large myocardial territory are possible explanations of this phenomenon, which probably also explains the prognostic benefit of recanalizing CTOs. Interestingly, Valenti et al. recently reported that staged CTO revascularization in patients undergoing primary PCI is associated with improved one year (1.7 vs 12 % in non-attempted or failed CTO-PCI group, p 0.025) and three-year cardiac survival [28].

## Pathology of chronic total occlusions

The basic pathological feature of a CTO consists firstly of a proximal cap, which is often fibrotic or calcified and either of tapering type or not. The body of the occlusion in the majority of CTOs has some degree of neovascularization and is composed of loose or dense fibrous tissue, atheroma, calcified tissue and focal lymphocyte infiltrate [19, 21]. Sakakura et al. in a recent autopsy series revealed that negative occlusion remodeling was frequent and particularly increased in long-standing CTOs; shorter duration CTOs demonstrated abundant organized thrombus and large necrotic core and CTOs with CABG were severely calcified [29]. The distal cap of the occlusion, opacified via the collateral circulation during injection in most cases, appears to have more frequently tapered morphology than the proximal cap (78.9 vs 48.4 % in a recent series), facilitating distal wire entry with the retrograde techniques [29].

## Collateral circulation in chronic total occlusions

Collaterals are interarterial connections that provide blood flow to a vascular territory whose original supply is compromised. They develop driven by shear forces along the pressure gradient through the recruitment of pre-existing interarterial connections and preserve to a variable extent the integrity of the territory supplied by the obstructed vessel. Some of these connections may be preformed to such an extent that they are immediately recruitable during vessel occlusion [30] and may even prevent a myocardial infarction when an occlusion develops gradually; yet, the presence of viability is not a prerequisite for collateral development. Collaterals generally require 2–12 weeks to fully develop their functional capacity and mostly remain below angiographic resolution (200 μm) [31].

Rentrop et al. in 1985 described an angiographic grading system that does not rate the collaterals channels themselves, but their effect in filling the occluded arterial segment (grade 0 = no collaterals; grade 1 = side branch filling of the recipient artery without filling of the main epicardial artery; grade 2 = partial filling of the main epicardial recipient artery; grade 3 = complete filling of the main epicardial recipient artery) [32]. Werner et al. in 2003 introduced a novel classification of the collateral circulation, essential for the assessment of collateral access in contemporary retrograde techniques, that was validated with reference to physiologic measures of collateral function (CC grade 0 = no continuous connection between collateral supplying and receiving vessel; CC1 = threadlike continuous connection; CC2 = side branch-like connection; CC3 > 1 mm diameter of direct connection - not included in the original description) [31].

The functional supply of even angiographically well-developed collaterals to the occluded arterial segment is by definition suboptimal, with less than 10 % of the collateral channels achieving a normal coronary flow reserve during pharmacological stress [33]. The high prevalence of coronary steal in CTOs indicates that patients with even well collateralized CTOs may benefit from a revascularization [33].

CTO revascularization results in the regression of the collaterals through a process starting immediately after the re-establishment of antegrade flow and extending further many months after the revascularization procedure. Acute reocclusion would therefore lead to an acute coronary syndrome in most cases, as the recruitment of collaterals is not instantaneous in most patients [34–36].

## Rationale and indication for CTO recanalization

Relief of symptomatic ischemia and angina and improvement of prognosis are the ultimate goals of CTO revascularization. The problem with symptoms related to a CTO is their often-atypical presentation that, along with the chronic nature of the situation, may lead patients to adapt to their limited exercise capacity and often deny typical angina and only report dyspnea at higher exercise levels. Still, there is substantial evidence from non-randomized reports and prospective registries to relate successful CTO recanalization with improved angina-related quality of life, most pronounced in patients with a symptomatic state before PCI [37–40].

Randomized data to support improvement of Left Ventricle (LV) function following successful CTO recanalization are not available. A number of observational studies, reporting from an era prior widespread DES utilization, revealed LV functional improvement following successful CTO PCI, with the effect on regional function generally more pronounced than the effect on global LV function as assessed by Ejection Fraction (EF) [41–43]. In the case of documented ischemia related regional impairment, functional recovery starts within 1 to 4 weeks after revascularization and is usually extends through 3 months post the index procedure [44–46]. Imaging techniques are most suitable to detect the presence of hibernating or stunned but viable myocardium. Magnetic Resonance Imaging (MRI) is the gold standard to detect pharmacologically-induced wall motion changes, precisely assessing myocardial fibrosis, perfusion and viability [47]; subendocardial extent of the late gadolinium enhancement smaller than 50 % of the wall thickness and reversible perfusion deficit greater than 10 % of the total myocardial mass with myocardial nuclear perfusion are currently used as gold standards for viability and prognostically relevant ischemia.

The prognostic impact of successful CTO recanalization remains under debate. A number of non-randomized series report favorable long-term outcome and decreased need for CABG on patients undergoing successful CTO PCI, but generally reflect an era non comparable to today’s standard of treatment [5–7, 48–52]. Of note, the target vessel seems to affect the prognostic outcome with a recent series reporting improved long-term survival with successful PCI of a CTO in the LAD and the LCX, but not the RCA [53]. Multicenter randomized trials, such as DECISION-CTO (NCT01078051) and EURO-CTO (NCT01760083), have been launched to further elucidate the prognostic impact of CTO revascularization [17]. In anticipation of those studies’ results, the indications to revascularization of CTOs should not differ from the indications to revascularization of subocclusive lesions and can be defined based on a potential improvement of prognosis. There is a considerable amount of data supporting the revascularization of coronary lesions causing silent ischemia of more than 10 % of myocardial volume; this applies also to CTOs with documented ischemia and viability in the territory supplied by the occluded vessel [17, 54, 55]. Several studies reported the adverse impact of incomplete revascularization on subsequent clinical outcomes [56, 57]. In the Synergy Between Percutaneous Coronary Intervention with Taxus and Cardiac Surgery (SYNTAX) trial, angiographic complete revascularization was achieved in only 52.8 % of the PCI patients, with the presence of a total occlusion being the strongest independent predictor of incomplete revascularization [57].

The dimension of the occluded artery and the presence of other critically narrowed arteries weigh heavily in the decision to revascularize a CTO. Patients with poorly controlled anginal symptoms with medical therapy may also have indications to revascularization [58]. A prerequisite to meet this indication is the optimization of the dose and type of drugs, starting from beta-blockers, and the demonstration of objective evidence of ischemia. In theory, indications to surgery or angioplasty are based on the same criteria and the decision between one or the other is purely technical; surgical revascularization may be favored in the presence of left main coronary artery disease, complex triple vessel disease (especially in patients with comorbidities such as insulin-dependent diabetes, severe left ventricular dysfunction or chronic renal insufficiency), occluded proximal left anterior descending artery and multiple CTOs with a relatively low anticipated success rate [59].

## Lesion characteristics

Lesion characteristics play an important role in the likelihood of successful CTO recanalization. Several studies-nonreflecting contemporary CTO PCI practice- have consistently reported that increasing age of the occlusion, presence of calcium, presence of a non-tapered stump, excessive tortuosity of occluded vessels, long occlusion length, side branches at the occlusion entry, bridging collaterals, and lack of visibility of path in the distal vessel predispose to technical failure [50, 60–62]. Increasing operator experience along with antegrade technique evolution increased procedural success rates from 60–70 to 80 %, limiting at the same time the traditional predictive factors of failure to two: occlusion length and severe calcification. Morino et al. in 2011 introduced a lesion-related difficulty-grading tool, the J-CTO score, based on a large series of antegrade recanalizations in Japan [63]. Length greater than 20 mm, presence of a greater than 45° bend within the occlusion, presence of intralesional calcification, delineation of a stump at the proximal end are four angiographic parameters shown to influence the success rate and time requested for anterograde recanalization. With the addition of a fifth parameter derived from the clinical history, a previous failed attempt, it is possible to calculate the J-CTO score attributing to each of these parameters one point. “Easy” lesions with a score of 0–1 had a success rate of greater than 90 % (97.8 and 92.3 % respectively) and required a short time for wire crossing in most cases. Success progressively falls with an increased score with “difficult” -J-CTO score equal or greater than 3- lesions having a 73.3 % success rate and demanding a prolonged time for crossing [63].

Traditional predictive factors of failure during recanalization with conventional antegrade techniques do not apply for the retrograde techniques. Factors such as lesion length, severe calcification, severe tortuosity and side branch at the CTO site are not shown to predict failure with retrograde techniques. The only predictive factors o failure seem to be related to collateral circulation characteristics, such as the presence and quality of the collaterals, their continuity and tortuosity, their location in the septum or in the epicardium, the angle of the collateral anastomosis with the CTO vessel [64].

As the CTO PCI techniques driven by the technological advancements are evolving leading to continuously improving outcomes, it is inevitable that the same will happen with the predictive factors of success/failure.

Non-invasive imaging, in particular coronary Multi-Slice Computed Tomography (MSCT), can also assist the procedural planning by delineating the characteristics of the CTO. A considerable number of operators employ MSCT in order to assess: 1) the length and three-dimensional course of the occluded arterial segment; 2) the presence of calcium at the CTO; 3) the vessel size and remodeling (either positive or negative); and 4) the quality of the vessel distal to the CTO d shrinkage CTO [65–70].

Many studies have identified MSCT-related predictors of failure such as occlusion length, calcification, negative remodeling and vessel bending and shrinkage CTO [65–70]. There are even studies identifying MSCT parameters predictive of adverse long-term clinical outcomes after CTO PCI [24].

In the absence of randomized efficacy data and considerations related to patient radiation exposure, the consensus of the EuroCTO Club was that MSCT cannot be recommended for routine pre-procedural imaging for CTO PCI, but should be considered for complex CTO lesions with expected success rate <50 % and in cases of repeat procedures after initial CTO recanalization failure [17].

## CTO recanalization techniques

**Fig. 1:**
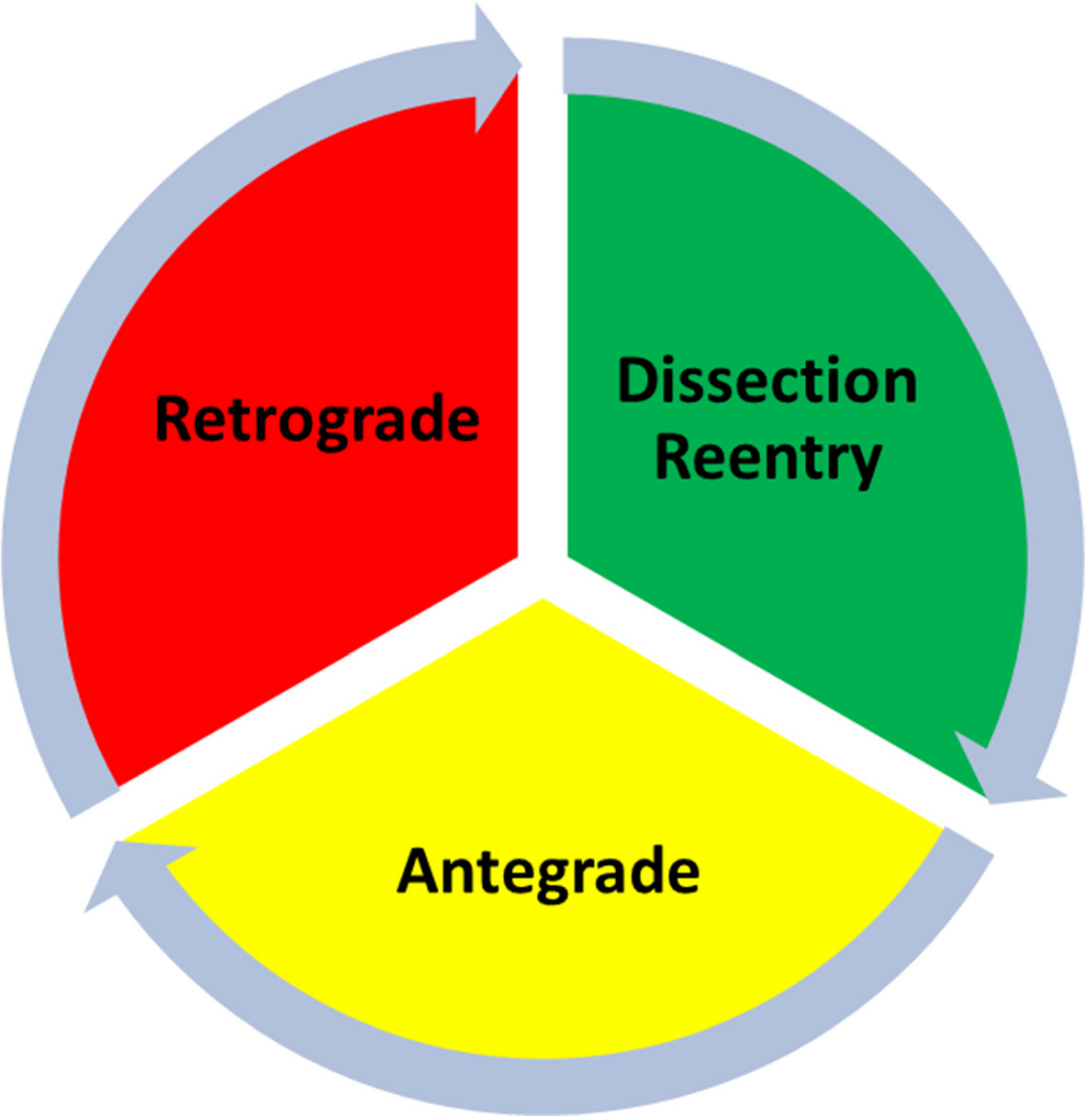
The continuum of CTO PCI

There are three basic CTO recanalization techniques: antegrade, retrograde and dissection reentry. In contemporary CTO treatment these techniques are complementary to each other and they can be alternatively applied and combined as necessary (Fig. 1).

**Fig. 2:**
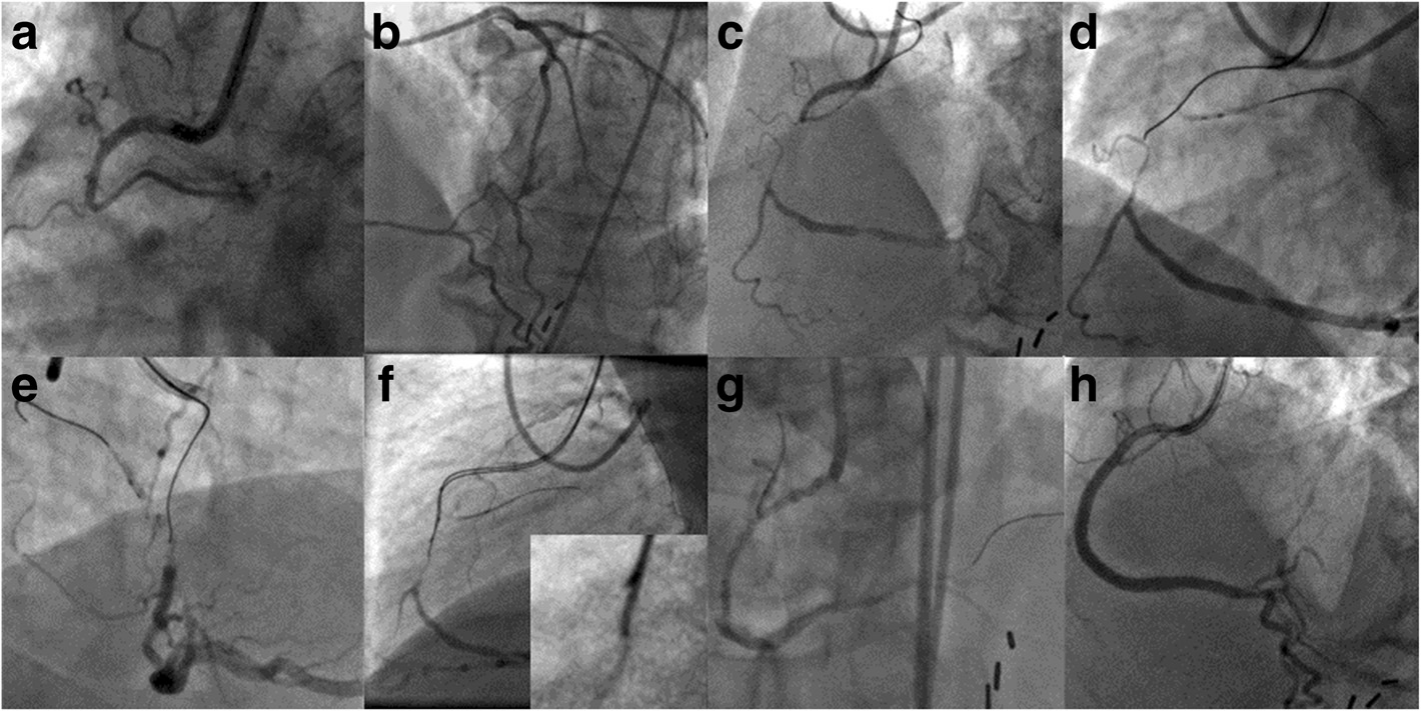
The Parallel Wire Technique. **a**: Right coronary artery (RCA) chronic total occlusion (CTO); blunt proximal stump and bifurcation at the CTO site. **b** Contralateral contrast injection revealing CC2 septal and epicardial collaterals from the Left Anterior Descending (LAD) coronary artery. **c** Bilateral contrast injection with the distal vessel opacified indicating a short and straight occluded segment. **d**, **e** To prevent dislodgment of the guiding catheter while advancing wire and microcatheter through the occlusion, a 2.5 × 20 mm balloon is inflated in an atrial branch proximal to the occlusion (anchoring technique). The wire (Fielder XT, Asahi Intecc, Japan) made progress through the body of the occlusion but appears to have deflected from the target. **f** A Confianza Pro 12 wire (Asahi Intecc, Japan) supported by a Corsair microcatheter (Asahi Intecc, Japan) is advanced towards the distal end of the occlusion parallel to the Fielder XT wire which is left in place. At the insert the distal segments of the two wires. **g** Successful chronic total occlusion (CTO) crossing; dissection at the site of the occlusion after predilatation. **g** Final angiographic result after implantation of 3.5 × 38 mm and 3.0 × 38 mm everolimus eluting stents with (Thrombolysis In Myocardial Infarction) TIMI III flow and no residual stenosis

Antegrade techniques are cornerstone and one way or another they are applied during a CTO recanalization procedure. It is widely accepted that they should be applied first. Antegrade single wire technique over a dedicated microcatheter is the simplest of all. There are two variations: the wire escalation approach (stiff-stiffer-stiffest) and the “step up-step down” approach (stiff-soft-stiff). The latter is used to accommodate hard or soft tissue penetration and the potential angulation of the occluded segment. In case of single wire technique failure, the parallel wire technique should be applied. The principle is to keep the first wire in place (subintimal space) and advance a second stiffer wire towards the distal true lumen using the first wire as a marker (parallel wire technique, Fig. 2).

**Fig. 3:**
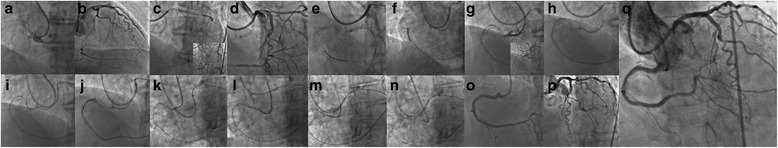
The Reverse Controlled Antegrade Retrograde Subintimal Tracking (CART) Technique. **a** Right coronary artery (RCA) with shepherd’s crook morphology occluded proximally; severely calcified chronic total occlusion (CTO) with blunt stump. **b** Contralateral injection revealing retrograde filling of the distal vessel via septal and epicardial collaterals. **c** After failure to cross the septal collaterals, switch to epicardial connections. Selective contrast injection through a Corsair microcatheter (Asahi Intecc, Japan) better delineates the course of the epicardial collaterals from the Left Circumflex Coronary (LCX) artery. **d**, **e** A tortuous continuous epicardial collateral (Werner CC2) is selected and crossed with a Fielder FC wire (Asahi Intecc, Japan). **f** Corsair microcatheter advanced into the distal true lumen over the Fielder FC wire; selective contrast tip injection confirms intraluminal position. **g** Retrograde wiring of the occlusion with a Gaia second wire (Asahi Intecc, Japan) and an Ultimate wire (Asahi Intecc, Japan); vessel course ambiguity. **h** Bilateral wiring of the occlusion with a Gaia second wire antegradely and an Ultimate wire (Asahi Intecc, Japan) retrogradely. **i** Retrograde wire knuckle in the subintimal space. **j** Antegrade balloon dilatations enlarging the subintimal space to facilitate retrograde wire crossing (reverse CART technique). **k** Retrograde wire (Ultimate) crosses the lesion and enters the ascending aorta; the Corsair is advanced through the body of the occlusion but the wire cannot be advanced in the antegrade guiding catheter. **l** Snaring of the retrograde wire into the antegrade guiding catheter. **m** Advancement of the Corsair into the antegrade guiding catheter. **n** Externalization of an RG3 wire (Asahi Intecc, Japan) allows antegrade insertion of balloons and stents. **o** Final angiographic result after implantation of 3.5 × 18 mm, 3.0 × 33 mm and 2.75 × 38 mm everolimus eluting stents. **p** Contralateral injection revealing intact collateral circulation. **o** Bilateral Injection confirming (Thrombolysis In Myocardial Infarction) TIMI III flow and no residual stenosis in the RCA

Retrograde techniques were revolutionized with the introduction of CART (Controlled Antegrade Retrograde subintimal Tracking) technique in 2005 by Katoh [71]. The novelty was the intentional collateral channel crossing, mostly septal, the channel dilatation with 1.5 mm balloons and the retrograde penetration and dilatation of the occlusion, most often close to the distal cap to facilitate antegrade wire crossing (CART). The main disadvantage was the need for septal dilatation with short balloons to allow passage of equipment resulting in complications with the collateral channels. The advent of dedicated microcatheters for collateral crossing, such as the Corsair from Asahi and the Finecross from Terumo, abolished the necessity for septal dilatation but also the capacity for retrograde dilatation of the occlusion, shifting the field to the Reverse-CART technique where balloon dilatation involved the body of the occlusion close to the proximal cap to facilitate retrograde wire crossing [16]. As of today this technique dominated the field and became the standard retrograde technique. In both techniques the main principle is the connection of the antegrade and retrograde subintimal spaces and the more recent development is the facilitation of this part of the procedure with the use of guiding catheters extensions (Guideliner, Vascular Solutions, Inc., Minneapolis, Minnesota), and Guidezilla, Boston Scientific, USA) with or without intravascular ultrasound (IVUS) use. After that, the externalization of a long 330 cm 0.010 in. (0.26 mm) diameter RG3 wire (Asahi Intec, Japan) is the final step in most of these complex procedures, providing excellent back-up support and allowing antegrade completion of the procedure (Fig. 3).

Dissection and reentry techniques are the evolutionary refinement of the STAR (Subintimal Tracking And Reentry) technique introduced by Colombo [71]. The principle of wire based dissection and reentry techniques is either antegrade or retrograde rapid steering of a stiff polymeric wire in the subintimal space, knuckled within the body of the occlusion. Knuckled wires tend to stay within the vessel architecture. These techniques are mainly used when there is vessel ambiguity within the occluded segment or whenever it is necessary for long arterial occluded segments to be crossed safely and fast. In certain occasions soft polymeric wires with small knuckles are used for dissection reentry aiming in minimal vessel disruption, mainly antegrade (mini-STAR technique). Device based dissection and reentry employ a dedicated device for antegrade subintimal space tracking (CrossBoss coronary catheter, Boston Scientific, USA) and distal wire exit is facilitated by a dedicated flat balloon with lateral ports (Sting-Ray, Boston Scientific, USA) [70].

Complimentary to these cornerstone techniques are adjunctive techniques such as balloon anchoring in side branches for active guiding catheter support and trapping of wires within guiding catheters to facilitate removal of long microcatheters [17]. Intracoronary imaging with IVUS and Optical Coherence Tomography (OCT) is also an important addition for the facilitation of the abovementioned techniques. The success rate in dedicated centers applying contemporary strategies remain in the field of 90 %, despite the escalating complexity of the lesions addressed over the last decade [68].

As of today the abovementioned techniques are well described but there is a lack of published universal definitions. There is also no universal consensus on how these techniques should be implemented and applied. The American school has proposed the Hybrid algorithm that describes a strategy of initial selection and rapid switching from antegrade to retrograde approach based on four simple questions related to CTO and collateral anatomy-proximal cap ambiguity, distal target, lesion length, and presence of interventional collaterals [72]. Such an algorithm is not widely adopted by European and even less by Japanese operators. Specifically the last ones consider dissection and reentry techniques non optimal approach for CTO recanalization, as there are considerations related to adverse clinical outcomes with their application, due to stenting of long subintimal spaces. There is lack of data in the literature related to the long term outcomes of CTO PCI with contemporary techniques that needs to be further addressed.

CTO recanalization techniques have evolved in parallel with the development of guidewire and microcatheter technology. The milestones of CTO guidewire development are presented in Table 1. The CTO wire toolbox with different types of dedicated wires and the indicated way of use is presented in Table 2 and other material CTO toolbox is presented in Table 3. One of the most frequently asked question is the sequence of wire selection in contemporary CTO techniques, both antegrade and retrograde. Such a question cannot be addressed based on individual wire suggestions as there is great variability in preferences even among the most experienced of the operators. What is important to be understood is the interaction of different kind of guidewires (soft < 1 gr, intermediate stiff 2–6 gr, stiff >9 gr) with different type of tissue (soft, hard and calcified). In general, soft (<1gr) tapered polymeric composite core guidewires are suitable for soft tissue tracking (passive wire control). Intermediate stiff (2–6 g) tapered composite core guidewires are used for hard tissue tracking (active wire control) and stiff (>9gr) tapered guidewires are suitable for calcified tissue penetration.

**Table 1:** Milestones in the CTO guidewire technology

Year	Milestone	Product
1995	Polymeric coating	SCIMED Choice PT
1996	Hydrophilic coating	TERUMO Crosswire
1997	Incremental tip load (drilling concept)	ASAHI Miracle family (3.5 up to 12 gr)
1998	Tapered Tip Design	GUIDANT HT CROSS-IT XT
1998	Combination of tapering with hydrophilic coating in high tip stiffness (>9 gr) (penetration concept)	ASAHI Confianza/Pro
2008	Combination of tapering with polymer and hydrophilic coating in low tip stiffness (<1 gr) (sliding concept)	ASAHI Fielder XT
2010/2011	Composite core tip in low tip stiffness	ASAHI SION/Fielder XTA/XTR
2013	Combination of composite core tapering polymeric and hydrophilic coating in intermediate stiffness (>1.5gr, <5 gr) (Deflection and rotation concept)	ASAHI GAIA family

**Table 2:** The CTO wire toolbox

Tapered, soft (~1) plastic jacketed GW (XT/XT-A/XTR)
➢ Antegrade/Retrograde microchannel/soft plaque probing
➢ Facilitation of quick wiring Dissection Re-entry in abmbigous vessel anatomy/soft plaque (Knuckle wire technique)
➢ Very small and tortuous collateral chanel crossing epicardial and septal(retrograde access)
Non-tapered, soft plastic jacketed GW (Fielder FC/Pilot 50/Whisper)
➢ Multi-tasting (Mainly work in the body of the occlusion-getting less fashionable)
Non tapered, medium gram force plastic jacketed wire (Pilot 150/200)
➢ Body of the occlusion
➢ Facilitation quick wiring in complex lesions and/or dissection-reentry in ambiguous vessel anatomy
Non-tampered, soft, composite core, hydrophilic coated CW (SION)
➢ Multitasking
➢ Access to difficult take-off collaterals
➢ Crossing of non challenging collaterals channels
➢ Subintinal spaces connection and GC engagement in retrograde techniques (CART/XCART)
Non-tapered, medium gram force (<6g), non coated, sliding wires (Miracle 3/4.5/6)
➢ Used to be workhorse wires for lesion crossing-tend to be abandoned
None-tapered, medium gram force (<6g), hydrophilic coated, sliding wires (Miracle Ultimate)
➢ For lesion crossing (body of the occlusion) in hard but not severely calcified plaques and non tortuous anatomy
Tapered, medium gram (<6g), composite core, hydrophilic coated GW (GAIA family)
➢ Are becoming the workhorse wires for lesion crossing (body of the occlusion) in the hard but not severely calcified plaques even in tortuous anatomy
➢ Subintima space connection in Retrograde techniques
Tapered and not tapered w-w/o hydrophilic coating, high gram (>9) GW penetration wires (Confianza fm, PROGRESS 200T)
➢ Crossing of severely calcified spots, exchanged to other categories afterwards

Fielder FC (Asahi Intecc, Japan), Fielder XT (Asahi Intecc, Japan), Fielder XTA (Asahi Intecc, Japan), Fielder XTR (Asahi Intecc, Japan), SION (Asahi Intecc, Japan), Gaia 1st/2nd/3rd (Asahi Intecc, Japan), Miracle 3/4.5/6 (Asahi Intecc, Japan), Miracle Ultimate (Asahi Intecc, Japan), Pilot 50/150/200 (Abbot Vascular, USA), Confianza family (Asahi Intecc, Japan), Whisper (Abbot Vascular, USA), Progress 200 T (Abbot Vascular, USA)

**Table 3:** Contemporary basic CTO PCI toolbox

Sheaths	45 cm long sheaths
Guiding catheters	7 and 8 Fr 90 cm long Guiding Catheters
Microcatheters	Corsair, Finecross, Venture, Tornus, Crusade, Multicross, Prodigy, Twinpass
Dissection and reentry	Crossboss coronary catheter, Stingray balloon
Guiding Catheter extensions	Guideliner, Guidezilla
Snares	Ensnare, Atrieve, Amplatz Gooseneck
Balloon uncrossable occlusions	Tornus, Laser, Rotational Atherectomy
Intravascular imaging	Intravascular Ultrasound (IVUS) (Eagle Eyes, Volcano, USA), Optical Coherence Tomography (OCT)
Complication managements	Jostent Graftmaster, Graftmaster Rx and Coil embolization
Radiation safety	Radpad
Stents	Drug Eluting Stents

Corsair (Asahi Intecc, Japan), Finecross (Terumo, Japan), Venture (Vascular Solutions), Tornus (Asahi Intecc, Japan), Multictoss (Roxwood Medical, USA), Prodigy (Radius Medical, USA), Crusade (kaneka, Japan), Twinpass (Vascular Solutions, USA), CrossBoss coronary catheter (Boston Scientific, USA), Sting-Ray (Boston Scientific, USA), Guideliner (Vascular Solutions, USA), Guidezilla (Boston Scientific, USA), Ensnare (Merit Medical, USA), Atrieve (Angiotech, USA), Amplatz Gooseneck, (Covidien, USA), IVUS (Εagle Eye, Volcano, USA), Jostent Graftmaster, Graftmaster Rx (Abbott Vascular, USA), RadPad (Worldwide Innovations & Technologies, USA)

## Operator requirements and CTO PCI complications

Opening complex CTOs remains a challenge requiring a certain learning curve before the operator becomes familiar and can be highly effective (>300 CTOs and >50 retrograde procedures per year), while simultaneously keeping the procedure safe; specific proctorship and guided training are indispensable elements in order to obtain the success rates reported above [72]. The incidence of complications remains low when these procedures are performed by experienced operators and high volume laboratories, despite the long procedural duration and use of multiple aggressive wires and catheters [73]. Major concerns are radiation exposure and contrast usage that are highly operator influenced and care should be taken to remain as low as possible [17]. Over long period of time CTO PCI complications rates remain <2 %, comparable to complication rates of non-occlusive coronary artery disease, and this seems to hold true also for advanced techniques and new dedicated guidewires [74–76]. Complications were significantly more likely to occur in the elderly, in women and in patients with triple vessel disease and depressed left ventricular function [74]. Wire exits and dissections are not uncommon in these procedures and are uneventful if promptly recognized and addressed. Drainage of pericardial tamponade and sealing of perforations with covered stents or microcoils are very rarely required but can be life-saving and the operator should be familiar with their use. Periprocedural myocardial injury, identified with systematic measurement of cardiac biomarkers post-CTO PCI, ensues in 8.6 % of patients, is more common with the retrograde approach, and is associated with worse subsequent clinical outcomes during mid-term follow-up [77].

## Conclusion

Technical and procedural success rates for CTO PCI have risen steadily over the last ten years, as a result of increased operator experience, improved materials, the refinement of the antegrade approach and the introduction of the retrograde approach, along with the development of dedicated groups of experts focusing on technical development, training and education worldwide. Data from large national registries still report underutilization of PCI for CTO, limited to 5–6 % of all the revascularization procedures and far below its prevalence [13, 78]. Further technical development is needed to facilitate and simplify the revascularization techniques, making them both safer and more standardized and predictable. Further evidence, ideally from randomized studies, of clinical benefit of these inherently complex procedures may encourage operators and centers to engage in this challenging endeavor.

## Abbreviations

CABG: coronary artery bypass grafting
CART: controlled antegrade retrograde subintimal tracking
CTOs: coronary chronic total occlusions
EF: ejection fraction
LAD: left anterior descending coronary artery
LV: left ventricle
MI: myocardial infarction
MRI: magnetic resonance imaging
MSCT: multi-slice computed tomography
PCI: percutaneous coronary intervention
RCA: right corοnary artery
STEMI: st segment elevation myocardial infarction
TIMI: thrombolysis in myocardial infarction
